# Cassava pulp added to fermented total mixed rations increased tropical sheep’s nutrient utilization, rumen ecology, and microbial protein synthesis

**DOI:** 10.5455/javar.2022.i645

**Published:** 2022-12-31

**Authors:** Pichad Khejornsart, Watcharawit Meenongyai, Theerayut Juntanam

**Affiliations:** Faculty of Natural Resources and Agro-Industry, Kasetsart University, Sakon Nakhon, Thailand

**Keywords:** Rumen fermentation, cassava pulp, TMR ensilage, feeding regimes, tropical lamb

## Abstract

**Objective:**

The price of animal production will be affected by the significant increase in feed costs. The objective of this research was to investigate the impact of adding waste cassava pulp to fermented total mixed ration (FTMR) on nutrient utilization, rumen ecology, and microbial protein synthesis in tropical sheep.

**Materials and Methods:**

A 3 × 3 replicated Latin square design was used to randomly arrange nine crossbreed lambs (Santa Inês × Dorper) with an initial body weight (BW) of 18.7 ± 1.6 kg (mean ± SD). During a 21-day trial, the animal was offered a random selection of concentrate diets and rice straw (control), total mixed ration (TMR), or FTMR. During the investigation, data on nutrition utilization, rumen ecology, and microbial protein synthesis were analyzed using analysis of variance.

**Results:**

The finding shows that FTMR had significantly higher dry matter (DM) intake and DM intake per BW than either TMR or control (*p *< 0.05). Lamb fed on FTMR had significantly higher DM, organic matter protein, crude protein, and neutral detergent fiber digestion than those on control or TMR (*p *< 0.05). Rumen pH values for all treatments ranged from 6.68 to 6.73, with no significant differences. Growing lambs fed FTMR had greater rumen total volatile fatty acid and propionic acid concentrations than those given TMR and the control (*p *< 0.05). Protozoa were not different across the FTMR groups, although total bacterial and fungal zoospores were increased. In addition, when lambs were fed FTMR containing cassava pulp, ruminal microbial protein synthesis was significantly increased.

**Conclusion:**

It could indicate that feeding growing lambs with FTMR could improve nutrient utilization, ruminal fermentation, and microbial protein synthesis. However, studies on the effects of FTMR on sheep performance, meat quality, and milk quality are necessary.

## Introduction

The price of animal feed has seen considerable changes worldwide, influencing the cost of farming livestock. Increases in feed prices ultimately cause increases in sheep production costs. For the future of agriculture and the distribution of animal protein, the rapid population growth along with the limited resources in many developing countries is a major problem [1]. Due to the cassava (*Manihot esculenta* Crantz) roots’ significance as a source of carbohydrates and the leaf’s significance as a supplemental source of protein in ruminants, it is grown in tropical and subtropical regions [2,3]. Cassava pulp has a high moisture content, primarily due to cassava starch production, and accounts for approximately 10%–15% of the raw root weight. Cassava starch manufacturers in Thailand were increasing their annual output to 1.5–2.2 million tons [4]. Fresh cassava pulp contains 15.8%–23.4% dry matter (DM), 2.2%–2.5% crude protein (CP), 55.0%–74.4% nitrogen-free extract, 17.9%–24.0% crude fiber, 74.4% total digestible nutrients, 12.9 MJ/kg DM digestible energy, and 11.3 MJ/kg DM metabolizable energy (ME) [5]. Recently, the price of cassava pulp is always cheap, approximately 250–550 THB/ton (1 USD = 32.63 THB). Consequently, cassava pulp is a potential feed resource to reduce the production cost of livestock. Pilajun and Wanapat [6] reported that the addition of molasses and urea enhanced the nutritional value of fermented cassava pulp with yeast and effective microorganisms, but not that of exogenous enzymes.

On ruminant animal farms, the feeding regimen has shifted from conventional to total mixed ration (TMR). The advantages of a TMR include higher feed intake, improved utilization of cheaper substitute materials for feed, a regulated forage concentrate ratio, and a decreased incidence of metabolic and digestive problems [7]. TMR is formulated in accordance with the animal’s total nutritional requirements and generally includes roughage (such as forage, agricultural wastes, and byproducts), concentrates, minerals, and vitamin types. TMR provides many benefits, but it also has major limitations, especially regarding feed sorting, that needs to be addressed [8,9]. Bhatt et al.’s [10] study in pre-weaning lambs found that the feeding method affected post-weaning weight. Moreover, TMR offered in block form emits less methane (CH4) and saves ME, besides improving CP digestibility, microbial protein synthesis, and nitrogen utilization [11]. TMR is a suitable form of feed, particularly when high-moisture agricultural byproducts are used [12].

Fermentation is the best way to store food because it is cheap and easy to make. It also keeps nutrients from going to waste and improves the feed [13,14]. Ensiling TMR has been shown to be an acceptable alternative to effectively managing wet byproducts in ruminant diets. It generally has significantly greater aerobic stability than the fresh TMR [15]. During recent years, there has been an improvement in the nutritive value and utilization efficiency of low-quality roughages and industrial by-products as TMR silage is efficient in overcoming this problem. TMR silage manufacturing is now widely used in Japan for the preservation and best use of high-moisture byproducts as ruminant feed [16]. As TMR silage fed with cassava pulp in growing lamb has not previously been studied, there is limited information now available on the various feeding regimens. Therefore, the objective of this study was to investigate what influence incorporating cassava pulp into TMR ensilage diets could have on sheep’s capability to use feed and the characteristics of rumen fermentation.

## Materials and Methods

### Animals and design

The research was undertaken at the Animal Science Research Farm of Kasetsart University, Chalermphrakiat Sakon Nakhon Province Campus (Northeastern region, Thailand; 17°17’N, 104 05’E). According to the Kasetsart University Guide for the Care and Use of Animals, the experimental approach was carried out in accordance with the necessary steps (Approval no. ACKU 60-ETC-016). Nine crossbred lambs (Santa Inês × Dorper) with an initial body weight (BW) of 18.7 ± 1.6 kg were numbered, dewormed, and randomly assigned into a 3 × 3 replicated Latin square design with 21-day each period. Each growing lamb was confined in a dedicated 1.2 × 0.8 m pen, with access to clean water and mineral blocks at all times. All animals were fed their treatments twice daily (8:00 a.m. and 16:00 p.m.).

### Experimental diets

The diet treatments included feeding the lambs separate concentrate diets containing 1.5% BW with rice straw offered as a control, TMR in which 50% roughage and 50% concentrate were used, or fermented total mixed ration (FTMR) during a 21-day experimental period. The machine set comprises a Kubota M7040 tractor with a power of 70 hp, which was used to mix TMR with 500 kg vertical single-auger TMR mixers. The TMR mixture was fermented for 21 days in the barrel before being fed to the animal. According to the Nutrient Requirements of Small Ruminants [17] for an average daily gain of 200 gm, Table 1 shows the percentage of ingredients in experimental diets.

### Data collection and laboratory analyses

Animal weights were taken throughout each trial period to regulate the feed intake and maintain a 10% remaining feed level in the trough. Daily samples of the feed and refuse were taken during the collecting period, and they were divided into periods before analysis. Samples of composites were ground through a 1 mm screen mash after drying at 60°C (Polymix® PX-MFC 90 D, Kinematica, Switzerland). All sample were then analysis for DM, organic matter (OM), and crude protein (CP) content [18], neutral detergent fiber (NDF), and acid detergent fiber (ADF) analysis using an ANKOM Fiber Analyzer (ANKOM Technology Corporation, Fairport, NY), which was adapted from the method of Van Soest et al. [19]. Fecal samples were obtained during the last 7 days by using rectal collection and analysis for AIA as the method of Van Keulen and Young [20] for studying nutrient digestibility. At the end of each period, rumen ﬂuid samples were collected after feeding at 4 h after feeding. A stomach tube connected to a vacuum pump collected approximately 200 ml of rumen fluid, which was then filtered through four layers of cheesecloth. The filtrate of ruminal fluid was immediately measured for pH (HANNA Instruments HI 9025, Singapore). The initial 50 ml of the mixture was put into a plastic container and mixed with 5 ml of 1 M H_2_SO_4_ before being kept at −20°C for ammonia-nitrogen (NH_3_–N) and volatile fatty acid (VFA) analysis. NH_3_–N concentrations in rumen fluids were determined using a Kjeltech Auto 1030 Analyzer [18], and ruminal VFAs were determined using high-pressure liquid chromatography (Agilent 1200 Series, Agilent Technologies Inc., Santa Clara, CA) with 0.1 M phosphate buffer as the mobile phase, as described by Samuel et al. [21]. The second portion, a 1-ml sample, was immediately added to 9 ml of 10% formaldehyde and stored at 4°C for total direct counts [22].

**Table 1.** Ingredients of concentrate, TMR, and FTMR used in the experiment.

Ingredient (% DM)	Concentrate	TMR	FTMR
Rice straw	-	40.0	40.0
Cassava chip	55.0	32.0	10.0
Cassava pulp	-	-	28.0
Rice bran	10.0	7.0	4.0
Soybean meal	9.0	4.5	4.0
Palm meal	10.0	7.0	6.0
*Leucaena* meal	9.0	5.5	4.0
Urea	1.0	0.5	0.5
Molasses	4.0	2.5	2.5
Sulfur	0.5	0.25	0.25
Salt	0.5	0.25	0.25
Mineral mix	1.0	0.5	0.5

According to Chen et al. [23], urine samples were taken twice daily for 2 days (morning and afternoon of the last 2 days of each period). The samples were then put into a plastic container with enough 50% H_2_SO_4_ to lower the pH to 2.5, and subsamples were immediately diluted and kept at −20°C for analysis. Purine derivatives (PD: uric acid and allantoin) and creatinine were evaluated in urine samples using HPLC. Based on purine derivatives: creatinine (PDC) index, PD excretion, and Please change to PD adsorption [24], the microbial protein synthesis was calculated as follows:

*Y* = 0.84*X* + (0.150 *W*^0.75^*e*^−0.25^*X*)

where: *Y*, PD excretion (mmol/day); *X*, PD absorption (once *Y* is determined, *X* can be calculated); 0.84, the recovery of absorbed purine as PD in urine (slope); 0.150, sheep value; *W*^0.75^, the metabolic BW (kg) of the animal; and *e*^ −0.25^, the endogenous contribution of PD to total excretion.

### Statistical analysis

An analysis of variance based on a 3* × *3 Latin square design was conducted using General Linear Models procedures of the Statistical Analysis System Institute [25]. Treatment means were compared by Duncan’s New Multiple Range Test with 5% significance (*p *< 0.05).

## Results and Discussion

### Chemical composition of experimental diets

The concentrate and TMR diets contained CP at 15.6%–15.9% DM and were formulated utilizing locally accessible feed resources. FTMR had cassava chips replaced with cassava pulp, and the DM content decreased as it increased (Table 2). The cassava pulp comprises 24.0% DM, 1.8% DM of CP, 92.4% DM of OM, 29.7% DM of NDF, and 15.3% of ADF. FTMR had cassava chips replaced with cassava pulp, and the DM content decreased as it increased. Ensiling is an aerobic crop preservation technique where the pH is lowered by the organic acids produced [14,27]. Furthermore, because of the high moisture content of cassava pulp, FTMR feed contained 65.6% DM, which is comparable with the findings of Kim et al. [7], who found DM to be 64% in TMR fermented. According to Hao et al. [26], TMR silages preserved for up to 56 days at different moisture levels (400, 450, and 500 gm/kg as fed) did not change in terms of their chemical composition, including IVDMD, NDF, soluble carbohydrates, CP, and ADF. However, Kondo et al. [28] showed that storage duration and temperature affected the quantities of soluble protein and NH_3_–N contained in TMR silage.

### Feed and nutrient utilization

When lambs were given FTMR, their total DM intake increased significantly (*p* < 0.05) (Table 3). The DM intake of FTMR in growing lambs was higher than that of those fed TMR and control (0.91, 0.76, and 0.72 kg/day, respectively). DM, OM, CP, and NDF digestibility were considerably greater in the FTMR group (*p* < 0.05), while ADF was unaffected. Total intake and digestibility were significantly higher in the FTMR group. It is possible to make every bite of feed practically a complete, nutritionally balanced meal for all cows using TMR instead of providing forages supplemented with concentrates. The decrease in rumen fill in response to pellets, which enables more feed to be consumed before reaching fullness, is the primary cause of the increase in DM intake [29]. Van Soest [30], on the other hand, stated that animal physiology and metabolic characteristics regulate and restrict feed consumption. One explanation for this effect might be that the rumen microorganisms are not receiving enough soluble growth nutrients from low intakes, such as energy and amino acids, or that the quality of feed fiber is limited. According to Kim et al. [7], a complete mix ration offers soluble growth factors that may promote the development of cellulolytic bacteria and cellulose digestion. Cereal crop silages have been shown conclusively to improve starch and protein digestibility depending on moisture content [31]. As the report of Bhatt et al. [10] shows, pre-weaning lamb showed a noticeably increased BW that provided adequate resources for the rumen microbe to accelerate fiber digestion, which might accelerate the passage and, in turn, increase feed intake and growth rate. Despite this, Yanti et al. [32] found that TMR silage had no impact on sheep intake, digestibility, rumen fermentation, or nitrogen balance. According to Chumpawadee and Leetongdee [33], goat intake, nutritional digestibility, ruminal fermentation, and chewing behavior were unaffected by feeding TMR mixed with cassava pulp. Additionally, Bueno et al. [15] observed that ruminants fed ensiled rations had an increase in feed efficiency as a result of the higher starch digestibility in TMR silages, including cereal grains. On the other hand, feeding sheep a complete feed block greatly enhanced their feed consumption and nutritional digestibility [29]. Jiwuba etal. [34] indicated that wet cassava pulp is low in quality protein and should be fermented as FTMR or with other rich protein sources before being used in ruminant diets.

**Table 2.** Chemical composition of concentrate, TMR, FTMR and rice straw used in the experiment.

Items	Concentrate	TMR	FTMR	Rice straw	Cassava pulp
**Chemical composition**					
DM, % of fresh matter	89.5	88.4	65.6	87.8	24.0
OM, % DM	90.3	89.5	90.1	88.6	92.4
CP, % DM	11.0	10.6	10.8	2.5	1.8
NDF, % DM	40.2	40.8	42.5	74.2	29.7
ADF, % DM	29.6	30.7	30.1	54.3	15.3

**Table 3.** Effects of difference feeding TMR ensilage containing cassava pulp on feed intake and appearance digestibility in growing lambs.

Items	Control	TMR	FTMR	SEM	*p*-value
**Feed intake**					
gm of DM	680.0^b^	850.0^a^	910.0^a^	29.036	0.034
gm/kg BW	37.82^b^	45.95^a^	46.67^a^	2.193	0.041
gm/kg BW^0.75^	77.15	80.35	94.90	6.956	0.109
Nutrients digestibility, %					
DM	64.83^b^	65.80^b^	68.48^a^	0.827	0.549
OM	68.97^b^	69.18^b^	70.64^a^	0.481	0.028
CP	56.28^b^	57.51^b^	58.90^a^	0.418	0.041
NDF	51.48^b^	52.25^b^	53.85^a^	0.513	0.045
ADF	37.55	38.03	39.23	0.658	0.482

Different letters on the row indicate significance at 5% (*p* < 0.05).

SEM: Standard error of the mean.

### Ruminal fermentation characteristics

Rumen fermentation parameters of lamb fed on different feeding regimes are shown in Table 4. Ruminal pH was not substantially different in any of the treatments (*p* < 0.05), which allowed us to examine the pattern of rumen fermentation. The rumen pH of the growing lamb that received FTMR was higher (6.73) than that of the animal that was given rice straw with concentrate separately (the control) and TMR (6.68 and 6.73, respectively). FTMR had higher rumen NH_3_-N concentration than the control and TMR (*p *< 0.05), whereas the control and TMR were not significantly different. The concentrations of total VFA and propionic acid were trending to be higher in the FTMR group than in the control group (*p* = 0.072). However, the proportions of acetic acid and butyric acid were unaltered. Ruminal pH values from this study ranged from 6.67 to 6.80, which was suitable for fermentation efficiency and microbial activities, as reported by Khejornsart et al. [35] and Khejornsart and Wanapat [36]. It was suggested that the fermentation mechanism might be caused by total mix ration silage, which comprises both non-structural carbohydrates and readily degradable carbohydrates. Furthermore, Cruywagen et al. [37] noticed that the operation of rumen ecology could only be affected if pH decreases below the 5.5 thresholds, at which rumen acidosis may be induced, rather than being lowered if pH does not rise beyond 6.1 on highly fermentable diets. FTMR had a higher rumen NH_3_-N concentration than control and TMR, whereas the differences between control and TMR were not significantly different. Bhatt et al. [11] found that lambs raised on grazing with concentrate supplementation with or without creep feeding did not show any effect of rumen NH_3_–N. It might be supported by the total VFA levels that Bhatt et al. [10] reported in the rumen fluid of developing lambs unaffected by feeding regimens. FTMR may be used in diets to improve the utilization of ruminal NH_3_–N for microbial protein synthesis due to the potential of substantial soluble carbohydrate components, such as starch and sugar. Khejornsart et al. [35] found a correlation between feed intake, digestibility, and the quantity of fiber in the diet. Additionally, feeding pelleted TMR raised the content of total short-chain fatty acids and lowered rumen pH [38].Cassava pulp has been shown to have good *in vitro* ruminal fermentation performance and organic matter degradability [39].

**Table 4.** Effects of difference feeding FTMR containing cassava pulp on rumen fermentation parameters of growing lambs.

Items	Control	TMR	FTMR	SEM	*p*-value
pH	6.65	6.68	6.73	0.750	0.627
NH_3_–N	14.11^b^	14.71^b^	15.40^a^	0.387	0.018
Total VFA, mmol	95.78^b^	101.10^a^	105.78^a^	1.440	0.025
VFAs, mol/100 ml					
Acetate (C2)	65.09	66.51	65.79	1.016	0.570
Propionate(C3)	21.87	21.34	22.39	0.652	0.072
Butyrate (C4)	12.94	12.15	11.83	1.041	0.528
C_2_:C_3_	3.09	3.12	2.94	0.317	0.104

Different letters on the row indicate significance at 5% (*p* < 0.05).

SEM: Standard error of the mean.

### Rumen microorganism population

Table 5 shows how different feeding patterns affect the ruminal microbe population in growing lambs. As observed, the number of bacterial and fungal zoospores differed considerably between all treatments, with the total mix ration silage having the highest number. The feeding pattern has no impact on the number of protozoa. Compared to the control group, which had bacteria and fungal zoospore populations of 4.12 × 10^9^ and 1.86 × 10^4^ cells/ml, respectively, the FTMR groups had greater populations of bacteria and fungi (*p* < 0.05). The feeding regime did not influence the protozoa population, while the bacteria and fungi populations were higher in the FTMR groups. According to Khejornsart et al. [35] and Khejornsart and Wanapat [36], chemical treatments enhanced the characteristics of rumen fermentation, increased the quantity of fibrolytic microbes and, consequently, the activities of fibrinolytic enzymes, and increased the number of sites that could support microbial attachment on the surface of the particles. According to Khejornsart et al. [40], there might be a link between plant phytochemical composition, particularly tannin and saponin content, and protozoa population reduction and methane mitigation. It appears that the roughage stimulated bacterial growth, enhanced feed particle colonization, and increased intake rate. This finding is significant because it demonstrates how FTMR improved the efficiency of rumen nutrient degradation, which increased the intake of cellulolytic bacteria by animals. Similarly, Khejornsart et al. [41] showed that treating rice straw with yeast enhanced animal intake, digestibility, rumen fermentation, and cellulolytic bacteria. According to Zhang et al. [42], feeding pelleted TMR to fattening lambs resulted in a shift in the composition of the rumen microorganisms. The rumen microbial population, however, was not altered at the phylum level by the TMR but did change marginally at the genus level [38].

**Table 5.** Effects of difference feeding FTMR containing cassava pulp on rumen microorganisms of growing lambs by using total viable count.

Items	Control	TMR	FTMR	SEM	*p*-value
Total bacteria, × 10^9 ^cell/ ml	4.12^b^	4.64^b^	6.19^a^	0.479	0.034
Protozoal, × 10^5^ cell/ ml	0.78	0.62	0.62	0.260	0.148
Fungal zoospore, 10^4^ cell/ ml	1.86^b^	2.08^b^	5.79^a^	0.315	0.020

Different letters on the row indicate significance at 5% (p < 0.05). SEM: Standard error of the mean.

### Microbial protein synthesis

Table 6 shows the impact of adding cassava pulp to FTMR feeding on the production of microbial proteins in growing lambs. The urine volume output of growing lambs with the control, TMR, and FTMR treatments were 980.0, 1,010.50, and 1,050.0 ml/day, respectively. Although there were no appreciable variations in the PD content between the TMR and FTMR treatments (*p* > 0.05), it was greater than in the control. Purine derivative absorption ranged from 7.63 to 13.27 mmol/day, microbial nitrogen supply ranged from 5.65 to 9.76 gm N/d, and the efficiency of microbial protein synthesis (EMPS) ranged from 20.13 to 25.09 gm N/kg digestible of organic matter in the rumen (DOMR). When given FTMR feed, these levels significantly increased (*p* < 0.01), while creatinine excretion did not differ between the treatments (*p* > 0.05).Suhartanto et al. [43] found that microbial protein synthesis, feed intake, and daily gain of goats when received TMR based on silage of sorghum were lower than fresh Napier grass. According to Nejad et al. [44], who found that the ESMN of Corriedale ewes fed commercial TMR varied from 6.10 to 7.53 gm N/day, the study‘s findings were comparable to theirs. The EMPS values in this study ranged from 20.13 to 25.09 gm N/kg DOMR. Numerous parameters, including DM intake, nitrogen ruminal degradability, carbohydrate sources, rumen dilution rate, mineral content, and other factors [45,46], affect the synthesis and production of microbial proteins. However, the PDC and PD excretion were unaffected (*p* > 0.05) by the various times of the spot urine sample [48]. The difficulty is in giving the option of utilizing the PDs and creatinine concentrations in a spot urine sample as well as the daily excretion of creatinine in urine as precise estimators of the PDs‘ excretion per day, which various diets offer. Even though this approach works well for comparing relative differences between treatments, absolute values should not be assumed.

**Table 6.** Effects of difference feeding FTMR containing cassava pulp on microbial protein synthesis.

Items	Control	TMR	FTMR	SEM	*p*-value
Creatinine excreted, mg/kg BW^0.75^	0.53	0.57	0.56	0.017	0.582
Urine output, ml/day	980.00	1,010.50	1,050.00	125.540	0.484
PD excreted, mmol/day	8.41^b^	12.32^a^	13.15^a^	1.512	0.042
PD absorbed, mmol/day	7.63^b^	12.29^a^	13.27^a^	1.204	0.030
Microbial nitrogen supply, gm N/day	5.65^b^	8.65^ab^	9.76^a^	0.904	0.010
EMPS, gm N/kg DOMR	20.13^b^	24.87^a^	25.09^a^	2.059	0.010

PD: purine derivative; EMPS, efficiency of microbial protein synthesis; DOMR: digestible of organic matter in the rumen (DOMI = organic matter intake—organic matter in fecal; DOMR = DOMI × 0.65; ARC, [47]); Different letters on the row indicate significance at 5% (*p* < 0.05). SEM: Standard error of the mean.

## Conclusions

Based on the findings of this study, it can be concluded that growing lambs given FTMR may consume more feed and digest nutrients more efficiently. Furthermore, the rumen fermentation parameters were higher in lambs fed concentrate with rice straw or TMR. Additionally, FTMR is simple for farmers to make and may be applied to sheep farms. It should be extensively advised to continue using TMR silage in lamb rearing.

## List of abbreviations

THB, Thai Baht, TMR, total mixed ration; FTMR, fermented total mixed ration; DM, dry matter; OM, organic matter protein; NDF, neutral detergent fiber; ADF, acid detergent fiber; BW, body weight; ME, metabolizable energy; PD, purine derivative; PDC, purine derivatives: creatinine index; EMPS, the efficiency of microbial protein synthesis; DOMR, digestible of organic matter in the rumen; CH4, methane; AIA, acid insoluble ash; VFA, volatile fatty acid; C2, acetate; C3, propionate; C4, butyrate.
